# Impact of Dry Sugar Beet Pulp on Milk Production, Digestibility Traits, and Blood Constituents of Dairy Holstein Cows

**DOI:** 10.3390/ani11123496

**Published:** 2021-12-08

**Authors:** Mohamed K. Mohsen, Mohamed F. Ali, Hamed M. Gaafar, Taha S. Al-Sakka, Salama M. Aboelenin, Mohamed M. Soliman, Mahmoud A. O. Dawood

**Affiliations:** 1 Animal Production Department, Faculty of Agriculture, Kafrelsheikh University, Kafr El-Sheikh 33516, Egypt; mohamedmahmoud201102@gmail.com (M.K.M.); mohamed.youssif3@agr.kfs.edu.eg (M.F.A.); tahasaka92@gmail.com (T.S.A.-S.); 2 Animal Production Research Institute (APRI), Agriculture Research Center, Ministry of Agriculture, Dokki, Giza 12619, Egypt; hamedgaafar@gmail.com; 3 Biology Department, Turabah University College, Taif University, Taif 21944, Saudi Arabia; s.aboelenin@tu.edu.sa; 4 Clinical Laboratory Sciences Department, Turabah University College, Taif University, Taif 21944, Saudi Arabia; mmsoliman@tu.edu.sa

**Keywords:** dry sugar beet pulp, Holstein cows, digestion, rumen fermentation, milk yield, feed conversion

## Abstract

**Simple Summary:**

There are many agriculture by-products in the agriculture sector, and many of them have high socio-economic value. There is a growing interest in recycling agro-industrial by-products for feeding ruminants. Sugar beets are used to produce sugar and result in sugar beet pulp by-products, which can be used as feed for livestock, either as dried flakes or as compressed pellets. In this study, the effect of partial replacement of yellow corn grains (YCG) with dry sugar beet pulp (DSBP) at levels of 25 and 50% was evaluated with respect to the milk production and health condition of dairy Holstein cows. Partial replacement of YCG with DSBP in the rations of dairy cows led to significant improvements in the digestion, rumen activity, plasma biochemical parameters, milk yield, milk composition, feed use, and milk yield. Furthermore, the reduction in feed cost and the increase in milk yield improved with increasing DSBP in the ration. Thus, replacing feed ingredients with DSBP is recommended for feeding dairy Holstein cows with low-cost feeds without reducing their health status and production.

**Abstract:**

Thirty multiparous lactating Holstein cows with an average live body weight of 642 ± 21 kg and an average daily milk yield of 30.46 ± 0.59 kg were used in this study. Cows with parities of 2 and 4 were used following their peak period, and were divided into three groups, with ten cows in each group. The control group was fed yellow corn grain rations (YCG), while for the 2nd and 3rd groups, 25 and 50% of YCG was replaced with dry sugar beet pulp (DSBP), denoted as DSBP_25_ and DSBP_50_, respectively. The contents of dry matter, organic matter, ether extract, nitrogen-free extract, and fiber carbohydrate in the experimental rations tended to decrease; however, crude protein, crude fiber, ash, and fiber fractions tended to increase in the DSBP_25_ and DSBP_50_ groups. Only crude fiber digestibility increased (*p* < 0.05) in the DSBP rations. Rumen pH value and concentration of ammonia nitrogen (NH_3_-N) decreased, while the concentration of total volatile fatty acids (TVFAs) increased in the DSBP_25_ and DSBP_50_ groups. The concentrations of total protein and globulin in blood plasma were higher (*p* < 0.05) in DSBP_25_ and DSBP_50_ than in YCG. However, plasma albumin concentration, aspartate aminotransferase (AST), and alanine aminotransferase (ALT) activities were lower (*p* < 0.05) in DSBP_50_ than in YCG. Milk yield and yield of 4% fat-corrected milk (4% FCM) were higher (*p* < 0.05) in DSBP_25_ and DSBP_50_ than in YCG. Fat, protein, solids not fat (SNF), and total solids (TS) contents in milk increased significantly (*p* < 0.05) for feeding rations containing DSBP. Feed cost was reduced, but the output of milk yield increased with DSBP. In conclusion, introducing DSBP into the rations of Holstein dairy cows led to significant improvements in their productive performance.

## 1. Introduction

It has become necessary to look for non-traditional feed ingredients in order to be able to provide the large amounts required for livestock [1]. The agri-food industry produces a large amount of waste and by-products characterized by high nutritional and economic value [2]. In Egypt, about 600,000 feddans (feddan = 4200 m^2^) were cultivated with sugar beet in 2018/2019, which produced about 715,600 tons of dried sugar beet pulp (DSBP). DSBP contributes 644,038 tons of dry matter (DM), 467,155 tons of total digestible nutrients (TDN), and 27,695 tons of digestible crude protein (DCP) [3] to the local nutrient supply. Fresh sugar beet pulp is dried using pulp dryer until it possesses approximately 10% moisture, and then it is pelleted to facilitate storage and transportation [4]. When sugar beets are processed into sugar, they produce sugar beet pulp as a by-product, which is used as feed for various livestock, either as dried flakes or as compressed pellets. Extracted grounded sugar beet pulp is used as ruminant feed and fed as fresh, dried, or silage (fermented beet pulp). Sugar beet pulp could be a highly palatable feed with moderate energy levels. It is low in sugar and different non-structural carbohydrates contents, containing fiber that is highly digestible, and necessary for ruminants to maintain rumen condition and increase acetate production [5].

Sugar beet pulp (SBP) is the solid substance produced during the extraction of sugar from sugar beets, representing about 6% of the DM of beet root weight [6]. Sugar beet pulp has a high crude fiber content (17–22%) and low crude protein content (8–11%) [7]. Despite this fact, it possesses excessive fiber, and its digestion is acceptable due to its low lignin content [8]. Consequently, SBP is considered to be an essential energy source in complete rations of dairy cattle and sheep [9].

An alternative of high-energy grains with such other feedstuffs as SBP is one means of lowering using excessive grains component in diets of ruminants [10]. Feeding Holstein cows on DSBP mid-lactation had a considerable effect on milk yield of about 10% (3–4 kg of milk) compared to the control group [11]. The high NDF content in SBP has a beneficial effect on milk yield and fat content [12]. Replacing 50% of energy ingredients (yellow corn, wheat bran, and rice bran) with DSBP in the rations of growing lambs improved their performance with respect to digestibility, rumen fermentation, and blood parameters, while increasing feed conversion and economic efficiency [13].

This work aimed to study the effect of partial (25 and 50%) replacement of yellow corn grains with DSBP on feed intake, digestibility, rumen fermentation, plasma parameters, milk yield, composition, and feed conversion in dairy Holstein cows.

## 2. Materials and Methods

The experimental work was carried out at a private farm of dairy cows located in West Delta, El-Beheira Governorate, Egypt, from January to April 2018. 

### 2.1. Experimental Animals

Thirty lactating Holstein cows with average live body weight 642 ± 21 kg, at 2nd and 4th lactation and average daily milk yield of 30.46 ± 0.59 kg were used at 60 days of lactation. According to live body weight, milk yield, and lactation, cows were divided into three similar groups (10 cows in each). The average live body weights of cows were 644, 643, and 639 kg, and the average milk yield at the beginning of the experiment was 30.42, 30.71, and 30.25 kg for cows in the three groups of the study. There were 3 cows in 2nd lactation, 3 in 3rd lactation, and 4 in 4th lactation in each group.

### 2.2. Experimental Rations

Cows of 1st group were fed the ration that contained Argentina yellow corn grains (YCG) and served as a control (YCG). While, in the 2nd and 3rd groups, 25 and 50% of YCG were replaced with Egyptian dry sugar beet pulp (DSBP) in DSBP_25_ and DSBP_50_, respectively. The intake of feed ingredients of the different rations is shown in Table 1. The compositions of ingredients and rations are shown in Table 2.

### 2.3. Management Procedure

Cows were housed in an open yard with a shed area corresponding to about one-third of the total area, and each cow occupied about 30 square meters on the yard floor and about 0.75 m at the feeding bunk. The floor was sandy dry due to exposure to direct sunlight all day round. The cows were fed total mixed ration (TMR) by group feeding in order to cover their recommended requirements for maintenance and milk production according to NRC [14] allowances for dairy cows. The rations were provided three times daily for 20 h a day and adjusted weekly according to changes in milk yield. Ample clean and cold water was available for the animals all day round.

### 2.4. Digestion Trials

Three digestion trials were conducted at the middle of the experiment for a seven-day collection period using three cows from each group. Rectum feces samples from each cow were taken twice daily at 12 h intervals, and samples of total mixed rations were taken during the collection period. Total mixed rations and representative feces sample composites were dried in a forced air oven for 48 h at 65 °C and then ground. Chemical analysis of samples of feedstuffs and feces were performed, consistent with the techniques of AOAC [15]. Digestibility coefficients were calculated using the equations of Schneider and Flatt [16], using insoluble acid ash (AIA) as a biological marker according to methods of Van Keulen and Young [17], as follows:

(1) $$\mathrm{Dry}\mathrm{matter}\mathrm{digestibility}\;\%\;=\;\;100-\left[100\;\times \;\frac{\mathrm{AIA}\;\%\;\mathrm{in}\;\mathrm{feed}}{\mathrm{AIA}\;\%\;\mathrm{in}\;\mathrm{feces}}\right]$$

(2) $$\mathrm{Nutrient}\;\mathrm{digestibility}\;\%\;=\;100\;-\left[100\;\times \;\frac{\mathrm{AIA}\;\%\;\mathrm{in}\;\mathrm{feed}}{\mathrm{AIA}\;\%\;\mathrm{in}\;\mathrm{feces}}\right]\;\times \;\left[\frac{\mathrm{Nutrient}\;\%\;\mathrm{in}\;\mathrm{feces}}{\mathrm{Nutrient}\;\%\;\mathrm{in}\;\mathrm{feed}}\right]$$

Fiber fractions were determined in rations, neutral detergent fiber (NDF), acid detergent fiber (ADF), and acid detergent lignin (ADL) [18]. Cellulose and hemicellulose were determined by the difference according to Rinne et al. [19], as follows:

Cellulose = ADF − ADL(3)

Hemicellulose = NDF − ADF(4)

Non-fibrous carbohydrate (NFC) was calculated according to NRC [14] as follows:

NFC = [100 − (NDF % + Crude protein % + Ether extract % + Ash %)](5)

Fiber fractions of ingredients and experimental rations are shown in Table 3.

### 2.5. Blood Samples

Blood samples were collected in heparinized clean test tubes via the jugular vein from 5 cows in each group. The samples were centrifuged for 10 min at 4000 rotations per minute to obtain plasma and kept frozen at −20 °C until chemical analysis. Total proteins, albumin, glucose, total lipids, triglycerides, and cholesterol and liver enzymes (alanine aminotransferase (ALT) and aspartate aminotransferase (AST)) were determined in plasma using kits (Diagnostic System Laboratories, Inc, Webster, TX, USA). Plasma globulin was calculated by subtracting the concentration of albumin from the total proteins. 

### 2.6. Rumen Fluid Samples

Samples of rumen fluid were taken from 3 cows of each group during digestibility trial by stomach tube attached to a vacuum pump at 4 h post feeding and strained through a double layer of cheese material [18]. Rumen pH was measured immediately using a virtual pH meter (Hanna gadgets pH, HANA^®^ instruments, Smithfield, RI, USA). Total volatile fatty acids (TVFAs) were determined using a steam distillation approach, as defined by Warner [20], and ammonia nitrogen (NH_3_-N) concentration was determined using magnesium oxide [15].

### 2.7. Milk Production and Samples

Cows were mechanically milked three times each day at 8 h intervals (at 5 a.m., 1 p.m., and 9 p.m.). Milk yield was recorded automatically for every cow through a digital milk meter fixed in every milking unit within the parlor, and the data were transported through the network to a computer system that computed the average milk yield per cow per day. Keeping with the week, month, and lactation season (the Afikim-herd management system), milk samples were taken during the 4th week of every month from the three milking times and composited proportionally to the milk yield of each cow. Samples were analyzed for fat, protein, lactose, solids not fat (SNF), and total solids (TS) by the infrared spectrophotometry (Foss 120 Milko Scan, Foss Electric, Hillerød, Denmark). We calculated 4% fat corrected milk (4% FCM) according to the formula of Gaines [21]: 4% FCM = Actual milk yield (kg) × 0.4 +15 × fat yield (kg).

### 2.8. Feed Utilization

Feed utilization efficiency in terms of dry matter (DM), total digestible nitrogen (TDN), crude protein (CP), and digestible crude protein (DCP) required for one kg 4% FCM yield were calculated for every cow as follows:

DM (kg/kg 4% FCM) = DMI/4% FCM yield(6)

TDN (kg/kg 4% FCM) = TDNI/4% FCM yield(7)

CP (g/kg 4% FCM) = CPI/4% FCM yield(8)

DCP (g/kg 4% FCM) = DCPI/4% FCM yield(9)

### 2.9. Economic Efficiency

Feed cost/kg and 4% FCM were calculated for every cow. Additionally, economic efficiency expressed as the ratio of 4% FCM yield and feed cost were calculated. Prices of one kg of ingredients (Egyptian pound, LE) of total mixed rations are shown in Table 4.

### 2.10. Statistical Analysis

The obtained results were statistically analyzed using the general linear models (GLM) procedure, adapted from the IBM SPSS Statistics [21] user’s guide, with one-way ANOVA according to the following model:

Y_ij_ = µ + x_i_ + e_ij_ (10)

where Y_ij_ = observation. µ = mean, x_i_ = the effect of treatment, e_ij_ = experimental error.

The degree of significance between the means of treatments was determined using Duncan’s test in the SPSS program. *p* < 0.05 was adopted as denoting statistically significant differences among the groups.

## 3. Results

### 3.1. Nutrient Digestion and Feeding Values

The nutrient digestion and feeding values for the experimental rations are presented in Table 5. Only CF digestibility was significantly higher (*p* < 0.05) with the introduction of DSBP (in DSBP_25_ and DSBP_50_) than that of YCG. However, the digestibilities of other nutrients was not significantly affected (*p* > 0.05) by DSBP, and were nearly the same for the different rations. 

The intake of DMI, TDNI, CPI, and DCPI were almost the same in different rations, with no significant differences, as shown in Table 5, with the cows group being fed each experimental ration as a total mixed ration (TMR).

### 3.2. Rumen Fermentation Parameters

The effect of replacing YCG with DSBP on the rumen fermentation parameters of cows is presented in Table 6. The ruminal pH values were significantly lower (*p* < 0.05) in DSBP_25_ and DSBP_50_ than in YCG. Additionally, ruminal NH_3_-N concentration was significantly lower (*p* < 0.05) in DSBP_50_ than in YCG, while DSBP_25_ was in between, with insignificant differences. However, concentration of ruminal TVFAs was substantially (*p* < 0.05) higher with the rations containing DSBP (DSBP_25_ and DSBP_50_) in comparison to the control ration (YCG).

### 3.3. Plasma Biochemical Traits

The plasma biochemical parameters of cows fed experimental rations are shown in Table 7. Introducing DSBP into the rations of dairy Holstein cows led to significant (*p* < 0.05) improvements in blood plasma biochemistry. Plasma total protein and globulin concentrations were significantly higher (*p* < 0.05) in DSBP_25_ and DSBP_50_ than in YCG. However, plasma albumin was significantly higher (*p* < 0.05) in YCG than DSBP_50_, while DSBP_25_ presented no significant difference. 

Introducing DSBP in the rations of dairy cows led to markedly (*p* < 0.05) reduced liver enzyme activity (AST and ALT). The values of AST and ALT were greatly (*p* < 0.05) higher in the plasma of YCG than of DSBP_50_, with insignificant differences being observed for DSBP_25_.

### 3.4. Milk Yield and Composition

The milk yield reported in Table 8 reveals that the yield of actual milk was higher significantly (*p* < 0.05) for cows fed DSBP rations (DSBP_25_ and DSBP_50_) than the control (YCG). At the same time, 4% FCM yield was greatly (*p* < 0.05) improved for DSBP_50_, followed by DSBP_25_, but YCG exhibited a decreased yield. Actual milk yield was increased by 1.55 and 3.05 kg/day or by 5.06 and 9.95% for DSBP_25_ and DSBP_50_ compared to YCG, respectively. The 4% FCM yield values were 5.70 and 10.98 kg/day or by 19.73 and 38.27%, respectively. The fact that the improvements in 4% FCM yield were more than those in the actual milk yield might be attributed to the improvements in the milk fat content.

The contents of fat, protein, SNF, and TS in the milk of cows increased significantly (*p* < 0.05) with feeding rations containing DSBP in addition to increasing the level of DSBP (Table 8). At the same time, lactose and ash contents were not affected significantly by DSBP (*p* > 0.05).

### 3.5. Feed and Economic Efficiency

The feed conversion ratio presented in Table 9 shows that cows fed with a high degree of DSBP (DSBP_50_) recorded significantly (*p* < 0.05) lower quantities of DM, TDN, and DCP per one kg four % FCM, followed those fed the low level of DSBP (DSBP_25_); however, higher amounts were detected for control feed (YCG). The improvements in feed conversion with the introduction of DSBP into the rations of dairy cows may be attributed to the increase of 4% FCM. 

Concerning the economic performance reported in Table 9, the average daily feed cost decreased with increasing DSBP level in the rations. While feed cost per 1 kg 4% FCM decreased significantly (*p* < 0.05) with increasing DSBP level in the rations, simultaneously, the average daily output of 4% FCM, net revenue, net revenue improvement, and economic efficiency increased significantly (*p* < 0.05) with increasing DSBP level in the rations of dairy cows. These outcomes indicate that the economic efficiency parameters improved appreciably (*p* < 0.05) with increasing levels of DSBP in the rations, which is probably due to the increase of the yield of 4% FCM.

## 4. Discussion

The comparison of chemical composition between yellow corn grains (YCG) and dry sugar beet pulp (DSBP) revealed higher DM, OM, EE, and NFE contents and lower CP, CF, and ash contents in YCG than DSBP. With respect to experimental ration, DM, OM, EE, and NFE contents decreased slightly; however, CP, CF, and ash increased slightly with increasing replacement level of YCG with DSBP. Similar results were obtained by Mahmoud and El-Bordeny [22] and Ali et al. [13]. The results of the fiber fraction revealed that the contents of NDF, ADF, ADL, cellulose, and hemicellulose were higher in DSBP than in YCG and increased in the rations with increased DSBP. On the other hand, non-fiber carbohydrate (NFC) was lower in DSBP than in YCG and decreased in the experimental rations with increasing DSBP. Similar findings were obtained by Mahmoud and El-Bordeny [22], who indicated that NDF, ADF, and ADL contents increased, but NFC decreased linearly with an increasing ratio of SBP in the rations.

The CF digestibility was significantly higher with the introduction of DSBP in DSBP_25_ and DSBP_50_ than in YCG. Similar results were obtained by Ali et al. [13], who found that the CF of beet pulp was highly digestible. Moreover, the degradability of the fiber fractions increased significantly with 10% DSBP supplementation in Italian ryegrass hay used for feeding fistulated goats [23]. Meanwhile, the digestibility of OM, NFE, and CP did not show any significant difference among the three rations containing 0, 17.13, and 34.25% DSBP. With respect to feeding values, the results showed that the TDN and DCP of the different rations were not significantly affected by replacing YCG with DSBP, and were nearly the same. Similar findings were reported by El-Badawi et al. [9], who found that TDN and DCP values differed insignificantly among rations when SBP replaced YC at 50 and 100% in lamb rations. Omer et al. [24] found that dietary treatments based mainly on concentrate feed mixture (CFM) composed especially of grains or primarily based on 90% DSBP plus 10% soybean meal as a fibrous concentrate did not affect TDN and DCP values. The nutritional value of DSBP could be fairly compared with that of excessive energy grains like corn, barley, or oats. The TDN value of DSBP was in the range of 68–74% [10].

The palatability and the bulkiness of dry sugar beet pulp can lend these characteristics to heavy concentrate mixtures, thus not affecting the feed intake [25,26]. Sugar beet pulp is high in rumen fermented energy (FME) in palatable shape [27]. Eweedah [28] showed that replacing YC with DSBP did not affect total feed intake by lambs. Abd-El Galil et al. [29] fed sheep on rations containing 70% CFM that contained 50% YC or SBP, and found no differences in total DM and CP intake between the experimental rations. Feed intake as dry matter, TDN, DCP, and DE was nearly the same for the control and DSBP rations [11].

Ruminal pH value was significantly lower in DSBP_25_ and DSBP_50_ than YCG. Additionally, ruminal NH_3_-N concentration was significantly lower in DSBP_50_ compared to YCG, while DSBP_25_ was intermediate, demonstrating insignificant differences. However, ruminal TVFA’s concentration was substantially higher in the rations containing DSBP (DSBP_25_ and DSBP_50_) in comparison to the control one (YCG). Total concentration of VFAs increased significantly with increasing proportion of DSBP in the ration [30]. A linear negative effect on ruminal pH and ammonia nitrogen concentrations with SBP rations has been reported compared with the control ration [22]. Rations containing DSBP have been reported to lead to substantially higher concentrations of TVFAs in the rumen fluid of lambs [13].

Introducing DSBP into the rations of dairy Holstein cows revealed significant improvements in blood plasma biochemistry. Plasma total protein and globulin concentrations were significantly higher in DSBP_25_ and DSBP_50_ than in YCG. However, plasma albumin was significantly higher in YCG compared to DSBP_50_, with no significant differences being recorded for DSBP_25_. In this respect, the groups fed DSBP had higher total protein and globulin values in serum than the control group in buffaloes and lambs [13].

Concentrations of glucose, total lipids, triglyceride, and cholesterol were almost comparable for the different groups and were not significantly affected by DSBP. Belibasakis and Tsirgogianni [31] reported no significant differences in the serum concentrations of glucose, triglycerides, and cholesterol when DSBP was added into the rations of Holstein cows, replacing and an equal proportion of YC.

Introducing DSBP into the rations of dairy cows led to a markedly low liver enzyme activity (AST and ALT). The values of AST and ALT were much higher in the plasma of YCG compared to that of DSBP_50_, with insignificant differences for DSBP_25_. The enzymatic activity of ALT and AST in the serum of cows in the control group was higher than the groups fed DSBP. The activity of ALT and AST was much better in the control group, while at the same time, both DSBP groups exhibited decreased values [13].

The investigation of yield revealed that the yield of actual milk was significantly higher for cows fed DSBP rations (DSBP_25_ and DSBP_50_) than the control group (YCG). At the same time, 4% FCM yield changed greatly for DSBP_50_, followed by DSBP_25_, but YCG had a decrease yield. Actual milk yield increased by 1.55 and 3.05 kg/day, or by 5.06 and 9.95% for DSBP_25_ and DSBP_50_ compared to YCG, respectively. The 4% FCM yield values were 5.70 and 10.98 kg/day or by 19.73 and 38.27%, respectively. The fact that the improvements in 4% FCM yield were greater than those in the actual milk yield might be attributed to the improvements in the fat content of the milk. The present results are in agreement with the findings of El-Badawi et al. [32], who found that daily milk yield increased significantly in cows fed the concentrate mixtures containing 25 and 40% DSBP compared to the control ration. Additionally, El-Fouly et al. [33] reported that daily milk yield was significantly increased in ewes fed the concentrate mixtures containing 27 and 54% DSBP compared to those fed the control ration. Petit and Tremblay [34] and Petit and Tremblay [35] fed Holstein cows on grass silage with ad libitum intake with a concentrated supplement containing soybean meal fed with corn (SBCO) or SBBP and reported that 4% FCM was higher by 3.5 kg/d for cows fed SBBP than those given SBCO-supplemented feed. El-Badawi et al. [32] found that the average daily yield of 4% FCM increased by 7.15% and 11.90% for cows fed concentrate mixtures containing 25 and 40% DSBP, respectively. El-Ashry et al. [8] stated that 7% FCM yield (kg/d) tended to increase with the increase of DSBP in buffalo rations.

The contents of fat, protein, SNF, and TS in cows’ milk increased significantly with feeding rations containing DSBP, in addition to increasing the level of DSBP. At the same time, lactose and ash contents were not significantly affected by DSBP. The improvements in milk composition when fed DSBP may be attributed to the higher CF, NDF, ADF, cellulose, and hemicellulose content of DSBP compared to YCG. Additionally, the improvements in milk composition detected in our study were directly attributed to the increase of fiber intake by cows and increased TVFA with increasing DSBP levels. Consistent with this observation, the fat content of milk increased for cows fed DSBP because of higher fiber intake and greater acetate concentrations in the rumen [36], and acetate is directly correlated with milk fat [37]. El-Fouly et al. [33] reported that milk fat content was significantly increased in ewes fed the concentrate mixtures containing 27 and 54% DSBP compared to the control ration. Preissinger et al. [38] stated that dairy cows fed DSBP had higher milk fat content. Mansfield et al. [39] found that milk fat percentage increased by 4.7% when beet pulp replaced corn in the ration of Holstein cows. Petit and Tremblay [34] and Petit and Tremblay [35] fed Holstein cows on grass silage ad libitum with a concentrated supplement containing soybean meal fed with corn (SBCO) or soybean meal fed with beet pulp (SBBP) and found that milk protein was higher with SBBP than SBCO. O’Mara et al. [40] found that milk protein was higher when feeding cows on concentrate based on beet pulp when using a concentrate based on corn or wheat. Mousa [41] reported that SNF yield in the milk of does fed a fodder containing beet roots was appreciably better than for those fed the control ration. El-Ashry et al. [8] found an increase in the total milk solids of buffalo fed DSBP compared to the control.

Feed conversion ratio showed that cows fed the high degree of DSBP (DSBP_50_) recorded lower quantities of DM, TDN, and DCP per one kg 4% FCM, followed by those fed the low level of DSBP (DSBP_25_); however, higher amounts were detected with the control ration (YCG). The improvements in feed conversion with the introduction of DSBP in the ration of dairy cows may be attributed to the increase of 4% FCM. Comparable results were obtained by Mansfield et al. [39], who found that efficiency of feed utilization was markedly improved in cows fed beet pulp compared with cows given corn. El-Badawi et al. [32] reported that feed conversion, either as DM or TDN/kg gain, was significantly improved with the ration containing 50% USBP compared to the control, but feed conversion deteriorated significantly when they were fed concentrate ration of 100% USBP. Talha et al. [6] stated that DM, TDN, or DCP/kg gain were better for lambs fed rations containing 50 or 75% DSBP than for those fed the control ration. The addition of 3% urea-treated sugar beet pulp to replace 50% of the common concentrate mixture is usually recommended in rations for growing sheep, and could provide a safe carbohydrate source with a more extended passage rate and, therefore, better utilization of dietary energy [42]. The incorporation of dried sugar beet pulp in the rations of growing lambs greatly improved feed conversion, with the lambs that were fed the control ration showing higher DM, TDN, DCP, and DE required per one kg live weight than those fed a ration containing DSBT [13].

The improved economic efficiency of feeding with DSBP is in agreement with the findings of Omer et al. [24], who found that the use of sheep ration using SBP supplemented with 10% SBM, replacing CFM, led to a decrease in the feeding price and improved the daily profit above the feeding price and feed cost LE/kg gain with respect to the control ration. Additionally, introducing SBP in the ration of sheep resulted in the formulation of a cheap ration, therefore reducing the feeding cost. Beet pulp is appropriate for use as a supplement for gestating or lactating cows, an ingredient in ideal diets, or as a replacement for roughage in finishing diets [41]. Feeding lambs on rations containing DSBP notably decreased the daily feed cost and feed cost per one kg weight gain, as well as increasing daily weight gain, net revenue, and economic efficiency compared with the control ration [11].

## 5. Conclusions

On the basis of these results, DSBP could be used as a partial replacement of yellow corn grains in the rations of dairy Holstein cows. The inclusion of DSBP led to significant improvements in feed intake, nutrient digestion, rumen fermentation, plasma biochemical parameters, milk yield and composition, feed conversion, and economic efficiency and increased with increasing dry sugar beet pulp in the ration.

## Figures and Tables

**Table 1:** Dietary ingredients for all the experimental diets.

Ingredient	YCG	DSBP_25_	DSBP_50_
Dietary Ingredients	% as Fed Basis		
Soybean meal	8.793	8.793	8.793
Yellow corn grains	17.885	13.414	8.943
Dry sugar beet pulp	0.000	4.471	8.942
Wheat bran	7.452	7.452	7.452
Alfalfa hay	14.904	14.904	14.904
Corn silage	35.770	35.770	35.770
Molasses	2.981	2.981	2.981
Megalac	0.835	0.835	0.835
Full-fat soybean	1.192	1.192	1.192
Premix	0.179	0.179	0.179
Sodium bicarbonate	0.536	0.536	0.536
Common salt	0.238	0.238	0.238
Limestone	0.075	0.075	0.075
Selenium	0.015	0.015	0.015
Chelated Mn	0.006	0.006	0.006
Chelated Zn	0.015	0.015	0.015
Life yeast	0.003	0.003	0.003
Nitro-tox	0.030	0.030	0.030
Calibrin-Z	0.149	0.149	0.149
Water	8.942	8.942	8.942
Total	100.000	100.000	100.000
Estimated nutritive value			
ME (Mcal/kg DM)	2.39	2.38	2.37
NEl (Mcal/kg DM)	1.50	1.49	1.49
CP (g/kg DM)	163.0	163.4	163.9
RUP (g/kg DM)	64.70	66.30	68.00
RDP (g/kg DM)	98.30	97.10	95.90

YCG: control group fed yellow corn grains (YCG) ration, DSBP_25_: diet with 25% dry sugar beet pulp (DSBP), and DSBP_50_: diet with 50% DSBP. ME: metabolizable energy, Nel: net energy for lactation, CP: crude protein, RUP: rumen undegradable protein, RDP: rumen degradable protein.

**Table 2:** Chemical composition of ingredients and experimental diets (g/kg).

Items	DM	OM	CP	CF	EE	NFE	Ash
Ingredients	g/kg						
Soybean meal	918.8	925.6	463.4	74.6	15.4	372.2	74.4
Yellow corn grains	907.0	984.8	89.2	19.4	42.2	834.0	15.2
Dry sugar beet pulp	880.7	953.5	98.7	246.7	6.0	602.1	46.5
Wheat bran	912.3	932.7	151.9	119.7	38.6	622.5	67.3
Alfalfa hay	932.1	875.9	195.2	276.2	17.0	387.5	124.1
Corn silage	297.6	917.0	76.4	314.9	15.7	510.0	83.0
Full-fat soybean	923.6	941.2	380.2	96.5	180.0	284.5	58.8
Molasses	781.0	902.0	44.0	4.1	1.0	852.9	98.0
Experimental diets	g/kg						
YCG	610.3	910.9	168.6	148.1	39.1	555.1	89.1
DSBP_25_	609.1	908.8	169.3	163.0	36.8	539.7	91.2
DSBP_50_	607.9	906.6	170.1	178.0	34.4	524.1	93.4

DM; dry matter, OM: organic matter, CP: crude protein, CF: crude fiber, EE: ether extract, NFE: nitrogen-free extract, YCG: control group fed yellow corn grains (YCG) ration, DSBP_25_: diet with 25% dry sugar beet pulp (DSBP), and DSBP_50_: diet with 50% DSBP.

**Table 3:** Fiber fractions of ingredients and experimental diets.

Item	NDF	ADF	ADL	Cellulose	Hemicellulose	NFC
Ingredients	g/kg					
Soybean meal	151.0	100.8	15.3	85.5	50.2	295.8
Yellow corn grains	88.2	28.9	7.1	21.8	59.3	765.2
Dry sugar beet pulp	433.0	273.4	20.8	253.2	159.6	415.8
Wheat bran	466.0	146.3	29.8	116.5	319.7	276.2
Alfalfa hay	450.2	321.8	101.9	219.9	128.4	213.5
Corn silage	470.5	272.0	27.9	244.1	198.5	354.4
Full-fat soybean	160.0	112.0	16.0	96.0	48.0	221.0
Experimental diets	g/kg					
YCG	279.1	174.2	34.6	139.6	104.9	403.3
DSBP_25_	303.9	182.0	36.1	145.9	121.9	371.0
DSBP_50_	328.8	189.9	37.6	152.3	138.9	338.5

YCG: control group fed yellow corn grains (YCG) ration, DSBP_25_: diet with 25% dry sugar beet pulp (DSBP), and DSBP_50_: diet with 50% DSBP. NDF: neutral detergent fiber, ADF: acid detergent fiber, ADL: acid detergent lignin, NFC: non-fibrous carbohydrate.

**Table 4:** Prices of ingredients of total mixed rations.

Ingredient	Price (LE)	Ingredient	Price (LE)
Soybean meal	7.87	Common salt	0.5
Yellow corn grains	4.16	Limestone	0.5
Dry sugar beet pulp	3.1	Selenium	140
Wheat bran	3.35	Chelated Mn	170
Alfalfa hay	3.2	Chelated Zn	120
Corn silage	0.5	Life yeast	20
Molasses	1.5	Nitro-tox	74
Megalac	18.5	Calibrin-Z	6.5
Full-fat soybean	8.2	Water	0.2
Premix	23.5	Milk	7
Sodium bicarbonate	7		

**Table 5:** Nutrient digestion and feeding values of experimental diets.

Item	Experimental Diets			SEM	*p*-Value
	YCG	DSBP_25_	DSBP_50_		
Digestion coefficients %					
DM	68.69	68.93	69.52	0.24	0.530
OM	72.02	72.55	72.75	0.17	0.621
CP	67.06	67.10	67.14	0.12	0.983
CF	67.06 ^b^	68.67 ^a^	69.42 ^a^	0.40	0.018
EE	74.68	74.73	75.17	0.20	0.808
NFE	75.26	75.78	75.99	0.27	0.681
Nutritive values %					
TDN	69.59	69.64	69.44	0.16	0.960
DCP	11.31	11.36	11.42	0.02	0.609
Feed intake (kg/day)					
Total DMI	20.47	20.43	20.39	0.12	0.972
TDNI	14.25	14.23	14.16	0.08	0.921
CPI	3.45	3.46	3.47	0.02	0.940
DCPI	2.32	2.32	2.33	0.01	0.955

^a,b^: Means in the same line with different letters differ significantly at 5% level. YCG: control group fed yellow corn grains (YCG) ration, DSBP_25_: diet with 25% dry sugar beet pulp (DSBP), and DSBP_50_: diet with 50% DSBP. DM: dry matter, OM: organic matter, CP: crude protein, CF: crude fiber, EE: ether extract, NFE: nitrogen-free extract, TDN: total digestible nitrogen, DMI: dry matter intake, TDNI: total digestible nitrogen intake, CPI: crude protein intake, DCPI: digestible crude protein intake.

**Table 6:** Rumen fermentation parameters of cows fed experimental diets.

Item	Experimental Diets			SEM	*p*-Value
	YCG	DSBP_25_	DSBP_50_		
pH value	6.92 ^a^	6.75 ^b^	6.67 ^b^	0.05	0.032
NH_3_-N (mg/100 mL)	16.29 ^a^	15.36 ^ab^	14.35 ^b^	0.39	0.023
TVFA’s (meq/100 mL)	25.33 ^b^	35.73 ^a^	36.40 ^a^	2.39	0.012

^a,b^: Means in the same line with different letters differ significantly at 5% level. YCG: control group fed yellow corn grains (YCG) ration, DSBP_25_: diet with 25% dry sugar beet pulp (DSBP), and DSBP_50_: diet with 50% DSBP, NH_3_-N: ammonia nitrogen, TVFA’s: total volatile fatty acids.

**Table 7:** Plasma biochemical parameters of cows fed experimental diets.

Item	Experimental Diets			SEM	*p*-Value
	YCG	DSBP_25_	DSBP_50_		
Total protein (g/100 mL)	7.67 ^b^	7.87 ^a^	7.90 ^a^	0.08	0.041
Albumin (g/100 mL)	3.17 ^a^	3.07 ^ab^	2.97 ^b^	0.05	0.038
Globulin (g/100 mL)	4.50 ^b^	4.80 ^a^	4.93 ^a^	0.12	0.034
Glucose (mg/100 mL)	65.43	66.15	66.72	0.76	0.784
Total lipids (mg/100 mL)	294.00	292.33	296.33	29.60	0.865
Triglyceride (mg/100 mL)	29.00	28.33	28.70	0.78	0.696
Cholesterol (mg/100 Ml)	160.00	158.90	158.33	8.86	0.891
AST (U/L)	111.67 ^a^	109.00 ^ab^	106.06 ^b^	2.83	0.046
ALT (U/L)	38.33 ^a^	35.00 ^ab^	31.33 ^b^	2.23	0.036

^a,b^: Means in the same line with different letters differ significantly at 5% level. YCG: control group fed yellow corn grains (YCG) ration, DSBP_25_: diet with 25% dry sugar beet pulp (DSBP), and DSBP_50_: diet with 50% DSBP, ALT: alanine aminotransferase, and AST: aspartate aminotransferase.

**Table 8:** Milk yield and its composition for cows fed experimental rations.

Item	Experimental Diets			SEM	*p*-Value
	YCG	DSBP_25_	DSBP_50_		
Average milk yield (kg/day)					
Actual milk yield	30.66 ^b^	32.21 ^a^	33.71 ^a^	0.34	0.036
4% FCM yield	28.69 ^c^	34.39 ^b^	39.67 ^a^	0.64	0.015
Milk composition %					
Fat	3.57 ^c^	4.45 ^b^	5.17 ^a^	0.11	0.017
Protein	3.05 ^b^	3.36 ^a^	3.45 ^a^	0.05	0.032
Lactose	4.79	4.80	4.82	0.04	0.415
Solids not fat (SNF)	8.55 ^b^	8.88 ^a^	8.96 ^a^	0.08	0.041
Total solids (TS)	12.12 ^c^	13.33 ^b^	14.17 ^a^	0.14	0.035
Ash	0.71	0.72	0.73	0.01	0.578

^a,b,c^: Means in the same line with different letters differ significantly at 5% level. YCG: control group fed yellow corn grains (YCG) ration, DSBP_25_: diet with 25% dry sugar beet pulp (DSBP), and DSBP_50_: diet with 50% DSBP.

**Table 9:** Feed efficiency for cows fed different rations.

Item	Experimental Diets			SEM	*p*-Value
	YCG	DSBP_25_	DSBP_50_		
Feed conversion ratio					
DM (kg/kg 4% FCM)	0.71 ^a^	0.59 ^b^	0.51 ^c^	0.02	0.012
TDN (kg/kg 4% FCM)	0.50 ^a^	0.41 ^b^	0.36 ^c^	0.01	0.017
CP (g/kg 4% FCM)	120.25 ^a^	100.61 ^b^	87.47 ^c^	2.21	0.021
DCP (g/kg 4% FCM)	80.86 ^a^	67.46 ^b^	58.73 ^c^	1.80	0.024
Economic efficiency					
Average daily feed cost (LE)	94.56	92.97	91.38	0.14	0.358
Feed cost LE/kg 4% FCM	3.30 ^a^	2.70 ^b^	2.30 ^c^	0.08	0.036
Average daily output of 4% FCM (LE)	200.83 ^c^	244.23 ^b^	277.69 ^a^	5.13	0.023
Net revenue (LE)	106.27 ^c^	151.26 ^b^	186.31 ^a^	5.24	0.016
Net revenue improvement (LE)	0.00 ^c^	44.99 ^b^	80.04 ^a^	5.12	0.011
Net revenue improvement (%)	0.00 ^c^	42.34 ^b^	75.32 ^a^	2.74	0.013
Economic efficiency	2.12 ^c^	2.63 ^b^	3.04 ^a^	0.06	0.018

^a,b,c^: Means in the same line with different letters differ significantly at 5% level. YCG: control group fed yellow corn grains (YCG) ration, DSBP_25_: diet with 25% dry sugar beet pulp (DSBP), and DSBP_50_: diet with 50% DSBP. DM: dry matter, CP: crude protein, TDN: total digestible nitrogen, DCP: digestible crude protein.

## Data Availability

The data presented in this study are available on request from the corresponding author.
